# Modeling the interplay between demography, social contact patterns, and SARS-CoV-2 transmission in the South West Shewa Zone of Oromia Region, Ethiopia

**DOI:** 10.1186/s12916-021-01967-w

**Published:** 2021-04-09

**Authors:** Filippo Trentini, Giorgio Guzzetta, Margherita Galli, Agnese Zardini, Fabio Manenti, Giovanni Putoto, Valentina Marziano, Worku Nigussa Gamshie, Ademe Tsegaye, Alessandro Greblo, Alessia Melegaro, Marco Ajelli, Stefano Merler, Piero Poletti

**Affiliations:** 1 Center for Health Emergencies, Bruno Kessler Foundation, Trento, Italy; 2 University of Udine, Udine, Italy; 3 University of Trento, Trento, Italy; 4 Doctors with Africa CUAMM, Padova, Italy; 5 Doctors with Africa CUAMM, Woliso, Ethiopia; 6 Doctors with Africa CUAMM, Addis Ababa, Ethiopia; 7 Dondena Centre for Research on Social Dynamics and Public Policy, Bocconi University, Milan, Italy; 8 Department of Social and Political Sciences, Bocconi University, Milan, Italy; 9 Department of Epidemiology and Biostatistics, Indiana University School of Public Health, Bloomington, IN USA; 10 Laboratory for the Modeling of Biological and Socio-technical Systems, Northeastern University, Boston, MA USA

**Keywords:** COVID-19, SARS-CoV-2, Mixing patterns, Contact data, Rural, Urban, Contact matrix, Transmission model, Epidemic

## Abstract

**Background:**

COVID-19 spread may have a dramatic impact in countries with vulnerable economies and limited availability of, and access to, healthcare resources and infrastructures. However, in sub-Saharan Africa, a low prevalence and mortality have been observed so far.

**Methods:**

We collected data on individuals’ social contacts in the South West Shewa Zone (SWSZ) of Ethiopia across geographical contexts characterized by heterogeneous population density, work and travel opportunities, and access to primary care. We assessed how socio-demographic factors and observed mixing patterns can influence the COVID-19 disease burden, by simulating SARS-CoV-2 transmission in remote settlements, rural villages, and urban neighborhoods, under school closure mandate.

**Results:**

From national surveillance data, we estimated a net reproduction number of 1.62 (95% CI 1.55–1.70). We found that, at the end of an epidemic mitigated by school closure alone, 10–15% of the population residing in the SWSZ would have been symptomatic and 0.3–0.4% of the population would require mechanical ventilation and/or possibly result in a fatal outcome. Higher infection attack rates are expected in more urbanized areas, but the highest incidence of critical disease is expected in remote subsistence farming settlements. School closure contributed to reduce the reproduction number by 49% and the attack rate of infections by 28–34%.

**Conclusions:**

Our results suggest that the relatively low burden of COVID-19 in Ethiopia observed so far may depend on social mixing patterns, underlying demography, and the enacted school closures. Our findings highlight that socio-demographic factors can also determine marked heterogeneities across different geographical contexts within the same region, and they contribute to understand why sub-Saharan Africa is experiencing a relatively lower attack rate of severe cases compared to high-income countries.

## Background

Despite limited access to healthcare [1, 2] and relatively milder social distancing restrictions compared to those imposed in most high-income countries [3, 4], coronavirus disease 2019 (COVID-19) mortality rates have been relatively low throughout Africa [5]. As of January 24, 2021, the World Health Organization (WHO) reports 2,462,083 diagnosed cases and 57,902 deaths in the continent [5]. However, severe acute respiratory syndrome coronavirus 2 (SARS-CoV-2) transmission dynamics have been highly heterogeneous across different African countries in terms of timing and implemented interventions [6].

In sub-Saharan Africa, Ethiopia is second only to South Africa in terms of the number of recorded cases and deaths, with an overall case fatality ratio (CFR) of about 1.5% compared to about 2.2% in the rest of the world [5]. The first COVID-19 case was confirmed on March 13, 2020, and, less than a month later, the Ethiopian Prime Minister declared a state of emergency in the country on April 8, 2020 [7]. Since then, rigorous contact tracing, isolation, and compulsory quarantine have been established [8, 9]. Borders and school closure were implemented, public institutions and firms operated at minimum capacity or under complete closure, and people were advised to stay at home [8]. However, in November 2020, schools reopened in the entire country, and social gatherings were allowed again. As of January 24, 2021, 133,298 SARS-CoV-2 infections and 2063 deaths [5] were ascertained in the entire country, with thousands of cases reported in all the 12 regions of Ethiopia [9]. In Ethiopia, a syndromic surveillance is carried out to identify SARS-CoV-2 infected individuals. Samples from suspected cases and case contacts are collected at different health facilities displaced in the country (including health centers serving the most rural areas) and cases are confirmed via real-time reverse transcription–polymerase chain reaction (RT-PCR) test. Collected samples are analyzed by 38 national, regional, hospital, and private laboratories [10]. Both suspected and laboratory-confirmed cases are admitted to isolation centers and discharged after a negative laboratory test [9]. Although swab testing was initially applied to both symptomatic patients and all close contacts of cases, it is possible that, due to limited resources and the increased number of cases in the country, only symptomatic case contacts are currently tested. Active monitoring of cases conducted by the Ethiopian Public Health Institute suggested that 52% of the identified positive cases were asymptomatic [11]. As of January 10, 2021, the overall rate for positive laboratory test results since the first detection of the epidemic in the country was 6.9% [9].

The possible spread of SARS-CoV-2 in rural areas of the country is especially dangerous because of the sparse presence of well-resourced health facilities implying long travel distances for remote populations, which is an important barrier to universal access to primary care [2]. Moreover, the healthcare workforce in Ethiopia is 5 times lower than the minimum threshold defined by the WHO for Sustainable Development Goals health targets [12] and far below the African average [13].

Recent modeling studies investigated the impact of control measures, such as self-isolation and temporary lockdowns, in a number of sub-Saharan African countries, highlighting the difficulties in defining effective, feasible, and sustainable strategies for suppression or mitigation of COVID-19 epidemics [14–17]. In this work, we aim to assess how demographic factors and age-specific mixing patterns can influence the impact of COVID-19 epidemics across different geographical contexts of the South West Shewa Zone (SWSZ) of the Oromia Region of Ethiopia, characterized by different levels of access to healthcare. So far, 21,133 cases were reported in the Oromia Region. The interventions implemented to control the epidemic were part of the national strategy designed by the Ministry of Health targeting all districts of the country, including the SWSZ. National measures undertaken between April and mid-September 2020 included the suspension of teaching activities at schools and universities. More stringent measures, including interruption of economic activities, restrictions on the use of public transport, and social gatherings (churches, mosques, markets, etc.), were partially adopted as well [8].

## Methods

### Study design

We conducted a survey based on individual interviews to estimate age-specific mixing patterns in four districts (*woreda*) of the SWSZ. About 40% of the SWSZ population is below 15 years of age and about 68% lives in remote rural settlements, 18% in rural villages, and 14% in the largest town of the area (Woliso Town, 53,065 inhabitants). The districts targeted by our study encompass a population of 449,460 inhabitants and represent the main catchment area of the St. Luke Hospital located in Woliso Town, a well-resourced health facility acting as the referral hospital for the entire Zone [2].

The study consists in a cross-sectional survey with two-stage stratified random sampling by location and age group. The survey was conducted in eight different sites, choosing two neighborhoods (*kebele*) for each district under study, in such a way to capture contact patterns in areas characterized by different population densities, work and travel opportunities, and access to the healthcare infrastructure. Three types of geographical contexts were considered: remote settlements (consisting of scattered subsistence farming settlements), rural villages (consisting of concentrated clusters of households served by a main road, and better access to main public services), and urban neighborhoods inside Woliso Town (significantly higher population density and full access to public services [18]).

For each site, a target sample size of 105 study participants was set on the basis of findings from previous contact surveys [19, 20] to provide the desired precision in the mean number of contacts (see Additional File 1: Sections 1 and 2 [20–22]). Households and study participants were randomly sampled using predefined quotas for each site, sex, and age group. A household was defined as a group of individuals living under the same roof and sharing the same kitchen on a daily basis. One individual per household was interviewed. If the study participant was a student, additional shorter interviews were performed to complement the data with information about close contacts occurring at school.

### Data collection

Participants were asked to recall information on the frequency, location, and type of social encounters from the day preceding their interview, providing the age (or age range when the exact age was unknown) and their relationship for each listed contact. A contact was defined as an interaction between two individuals, either physical (when involving skin-to-skin contact) or non-physical (when involving a two-way conversation with five or more words in the physical presence of another person, but no skin-to-skin contact) [19, 20]. The participants’ age, sex, education, and occupational status were recorded along with details on their household composition.

In the SWSZ, schools may host up to 100 students within a single class. To avoid inaccurate reporting of the number of school contacts, participants were only asked to count the total number of physical contacts they had at school in the previous day, without further details. Information on the age of students attending the targeted schools for different grades was also collected. Interviews were carried out between November and December 2019, i.e., prior to the COVID-19 pandemic. Schools were regularly open during the survey period.

### Contact patterns and data analysis

For each type of geographical context, we computed the mean number of contacts reported by respondents after grouping by age (six 10-year age groups from 0 to 59 years and one age group for individuals aged 60 years or older) and by contact setting (households, schools, and the general community). Since for many study participants it was difficult to distinguish encounters occurred because of their job from other random contacts, all social interactions occurring outside family and schools were aggregated with contacts occurring in the general community. Age-specific contact matrices were computed considering both physical and non-physical contacts and were adjusted for reciprocity as in [19]. Variability due to sampling of study participants was explored by computing 1000 bootstrapped contact matrices [23], where each bootstrap consisted in sampling with replacement a number of interviews equal to the original sample size, choosing the age of the participant with probability proportional to the Ethiopian age distribution [24]. The proportions of the SWSZ population living in remote settlements, rural villages, and in urban neighborhoods were used as sampling weights to compute an average contact matrix for the entire SWSZ. Full details about the study design, data collection, and the analysis of contact patterns are provided in the Additional File 1: Sections 1–7 and in the Additional File 2.

### Transmission model

We simulated SARS-CoV-2 spread in the SWSZ, using an age-structured Susceptible-Infectious-Recovered (SIR) compartmental model with three consecutive stages of infectiousness, in such a way to reproduce a gamma-distributed generation time of mean 6.6 days [25–27]. The model was run separately for each geographical context (i.e., the remote, rural and urban neighborhoods), using estimates of the population age structure and of the age-specific contact matrix computed from survey data (see Additional File 1: Sections 4–6). These data were collected in the absence of any restrictions imposed to control the infection spread. Because school closure in all of Ethiopia was mandated much before the exponential growth of reported COVID-19 cases, transmission of SARS-CoV-2 in the SWSZ was simulated by removing contacts occurring at school and considering only household and community contacts. In the model, 1000 values of the per-contact transmission rate were considered by matching the reproduction number computed through the next-generation matrix approach [28] with random samples from the posterior distribution of the reproduction number estimated from the curve of reported cases in Ethiopia during the phase of exponential growth [5, 29]. As the same public measures and restrictions were applied across different geographical contexts in Ethiopia, heterogeneous transmission of SARS-CoV-2 was assumed to be driven by differences in the demographic and contact structures in urban, rural, and remote neighborhoods. The same per-contact transmission rate was therefore assumed across different settings of the SWSZ and estimated using the sum of contact matrices obtained for the urban, rural, and remote neighborhoods, weighted by the percentage of SWSZ population living in each geographical context. We included school contacts to estimate the theoretical SARS-CoV-2 transmission potential in the absence of a school closure mandate.

We considered susceptibility to SARS-CoV-2 infection to vary with age. We adopted the posterior distributions estimated in Zhang et al. [23] for the relative probability of developing infection upon effective exposure to an infectious case, where the age group 15–64 years is taken as a reference; an average relative susceptibility of 0.33 (95% CI 0.24–0.47) was considered for children under 15 years of age and of 1.47 (95% CI 1.16–2.06) for older adults (above 65 years) [23]. These estimates are aligned with other independent studies (reviewed in Viner et al. [30]). We assumed the same infectiousness across individuals of different ages (see Additional File 1: Section 4 [31]).

We computed projections of the number of SARS-CoV-2 infections, cases with respiratory symptoms or fever, and COVID-19 critical cases (either requiring mechanical ventilation or resulting in a fatal outcome), based on available estimates of the age-specific risks [32]. By comparing estimates obtained when including and excluding school contacts for the entire duration of the epidemic, we computed the overall percentage of infections, symptomatic, and critical cases that could be averted by school closure.

To explore the robustness of our findings with respect to model assumptions, five separate sensitivity analyses were carried out assuming (1) a Susceptible-Exposed-Infectious-Recovered (SEIR) model structure, (2) a 20% increase or a 20% decrease of the net reproduction number, (3) different per-contact transmission rates across geographical settings, (4) homogeneous susceptibility by age, and (5) a lower infectiousness of children (see Additional File 1: Section 8). As the probability of developing symptoms after infection markedly increases with age [32, 33], the latter sensitivity is similar to exploring the effect of differential infectiousness among symptomatic and asymptomatic cases.

## Results

### Social contact data

A total of 938 study participants were interviewed with 43% of them living in rural remote settlements, 35% in rural villages, and 22% from urban neighborhoods (Table 1). Two hundred twenty-seven participants were students, 22.9% of whom were between 5 and 9 years of age, 71.8% between 10 and 19 years, and 4.9% older. School attendance rates among the study participants aged 5–18 years were 67%, 80%, and 77% in remote, rural, and urban sites, respectively. The median class size ranged from 70 children per class in rural villages to 90 in remote settlements. Only 27% of our study participants reported travels outside their village in the last month; 87.3% reported they were never admitted to the local hospital (see Additional File 1: Section 7).

**Table 1:** Characteristics of study participants and relative percentages in the Ethiopian population

	Number of study participants				
	Overall	Remote	Rural	Urban	
Variable*	***n*** (%)	***n*** (%)	***n*** (%)	***n*** (%)	Ethiopia (%) [24]
**Total**					
	938 (100.0)	400 (42.6)	326 (34.8)	212 (22.6)	–
**Age**					
< 10 years	382 (40.7)	160 (40)	137 (42)	85 (40.1)	27.3
10–19 years	198 (21.1)	85 (21.2)	66 (20.2)	47 (22.2)	24.1
20–29 years	92 (9.8)	40 (10)	32 (9.8)	20 (9.4)	18.4
30–39 years	117 (12.5)	50 (12.5)	42 (12.9)	25 (11.8)	12.0
40–49 years	59 (6.3)	26 (6.5)	18 (5.5)	15 (7.1)	7.9
50–59 years	40 (4.3)	17 (4.2)	13 (4)	10 (4.7)	4.9
60 years +	50 (5.3)	22 (5.5)	18 (5.5)	10 (4.7)	5.3
**Occupation**					
Pre-school	309 (32.9)	129 (32.2)	109 (33.4)	71 (33.5)	–
Student	226 (24.1)	85 (21.2)	87 (26.7)	54 (25.5)	–
Manual/office/shop worker	62 (6.6)	5 (1.2)	30 (9.2)	27 (12.7)	–
Housewife	137 (14.6)	66 (16.5)	47 (14.4)	24 (11.3)	–
Agriculture**	112 (11.9)	84 (21)	25 (7.7)	3 (1.4)	–
Unemployed/retired	44 (4.7)	9 (2.3)	12 (3.7)	23 (10.8)	–
Other	48 (5.1)	22 (5.5)	16 (4.9)	10 (4.7)	–
**Sex**					
Female	478 (51)	206 (51.5)	170 (52.1)	102 (48.1)	50.0
Male	460 (49)	194 (48.5)	156 (47.9)	110 (51.9)	50.0

* No missing data for any of the three listed variables

** The percentage of male adults (18–64 years old) working in agriculture is 45.2%; in the remote, the rural, and the urban settings, this percentage is 81%, 28%, and 7%, respectively

Age and sex were also recorded for all the 4635 household members of the 938 study participants. The mean household size in remote settlements was 5.5 (95% CI 5.3–5.7), significantly larger (Tukey test *p* < 0.001) than in rural villages (4.6, 95% CI 4.4–4.8) and in urban neighborhoods (4.4, 95% CI 4.2–4.6), while no significant difference in the household size was found between the latter two settings (Tukey test *p* = 0.48).

Overall, 5690 non-school contacts were reported by the 938 study participants (median 6 contacts per person, range 1–26, see Table 2). Of these, 79.9% were physical and 43.0% involved a single social interaction during the day.

**Table 2:** Mean number of recorded daily contacts, excluding contacts at school, by age, across different geographical contexts

	Mean number of contacts per day (excluding school contacts)			
Variable	Overall mean (95% CI)	Remote mean (95% CI)	Rural mean (95% CI)	Urban mean (95% CI)
**Total**				
	6.07 (5.88–6.26)	6.19 (5.87–6.51)	5.73 (5.44–6.02)	6.35 (5.96–6.73)
**Age**				
< 10 years	5.57 (5.32–5.83)	5.67 (5.23–6.12)	5.21 (4.84–5.58)	5.96 (5.46–6.47)
10–19 years	6.48 (6.02–6.94)	6.33 (5.6–7.06)	6.30 (5.63–6.98)	7.00 (5.96–8.04)
20–29 years	5.77 (5.28–6.26)	5.8 (5.04–6.56)	5.72 (4.84–6.6)	5.80 (4.82–6.78)
30–39 years	6.99 (6.41–7.57)	6.84 (5.96–7.72)	7.05 (6.00–8.09)	7.20 (6.05–8.35)
40–49 years	6.86 (6.08–7.65)	7.23 (5.85–8.61)	6.67 (5.51–7.82)	6.47 (5.01–7.92)
50–59 years	5.80 (4.90–6.70)	5.76 (4.66–6.87)	5.77 (3.95–7.59)	5.90 (3.74–8.06)
60 years +	5.84 (4.69–6.99)	7.73 (5.54–9.91)	3.56 (2.84–4.27)	5.80 (4.26–7.34)
**Sex**				
Male	6.15 (5.87–6.43)	6.15 (5.69–6.61)	6.02 (5.55–6.49)	6.34 (5.77–6.9)
Female	5.99 (5.73–6.24)	6.23 (5.79–6.67)	5.46 (5.11–5.82)	6.36 (5.84–6.89)
**Occupation**				
Pre-school	5.42 (5.17–5.66)	5.51 (5.11–5.91)	5.08 (4.72–5.44)	5.76 (5.24–6.29)
Student	6.57 (6.10–7.04)	6.51 (5.63–7.38)	6.24 (5.61–6.88)	7.20 (6.25–8.15)
Manual/office/shop worker	7.23 (6.34–8.12)	7.0 (3.72–10.28)	7.47 (6.06–8.87)	7.0 (5.78–8.22)
Housewife	5.76 (5.34–6.17)	5.67 (5.06–6.28)	5.43 (4.75–6.1)	6.67 (5.68–7.65)
Agriculture	7.02 (6.35–7.68)	7.35 (6.53–8.16)	5.92 (4.86–6.98)	7.00 (5.04–8.96)
Unemployed/retired	5.18 (4.4–5.96)	6 (3.88–8.12)	4.42 (3–5.83)	5.26 (4.25–6.27)
Others	5.83 (5.26–6.4)	6 (5.14–6.86)	5.69 (4.68–6.7)	5.7 (4.42–6.98)
**Setting**				
Household	2.8 (2.68–2.92)	2.94 (2.74–3.15)	2.48 (2.28–2.67)	3.02 (2.8–3.24)
Community	3.27 (3.09–3.45)	3.25 (2.95–3.54)	3.25 (2.97–3.53)	3.33 (2.98–3.67)
**Traveled to a different neighborhood in the month prior the interview**				
Yes	6.21 (5.83–6.59)	6.08 (5.34–6.81)	5.66 (5.16–6.16)	7.22 (6.46–7.97)
No	6.01 (5.79–6.23)	6.22 (5.86–6.57)	5.76 (5.4–6.13)	5.93 (5.51–6.35)
**Contacts outside neighborhood (%)**				
0–14 years	0.67% (0.4–1.04)	0.38% (0.14–0.94)	0.19% (0.03–0.77)	1.80% (1.03–3.09)
15–59 years	3.98% (3.23–4.89)	4.54% (3.36–6.07)	3.3% (2.19–4.9)	3.91% (2.47–6.07)
60 years+	2.74% (1.28–5.53)	1.18% (0.20–4.63)	0.00% (0.00–0.00)	10.34% (4.28–21.84)

For all sites, contacts outside school were predominantly reported between family members (46.1%), neighbors (25.2%), and other relatives outside the household (13.1%), while the remaining 15.5% of contacts occurred with friends, schoolmates outside school, or other unspecified categories. Individuals with a recent history of travel outside their neighborhood did not report an increased number of contacts, except for urban residents (*t* test *p* = 0.004). The mean number of contacts (excluding school contacts) reported by participants was lower in rural villages (5.73, 95% CI 5.44–6.02) with respect to both urban neighborhoods (6.35, 95% CI 5.96–6.73) and remote settlements (6.19, 95% CI 5.87–6.51). In particular, the mean number of daily contacts reported by the elderly (60+ years old) was much higher in remote settlements and urban neighborhoods than in rural villages (7.7 and 5.8 vs. 3.6, see Table 2).

Students reported 1372 additional contacts in schools, resulting in a mean number of 6.1 (95% CI 4.98–7.16) daily physical contacts per child (median 3, interquartile range 0–10). There were limited differences in the mean number of school contacts across geographical contexts (6.31, 95% CI 4.13–8.50 in remote settlements; 5.70, 95% CI 4.19–7.21 in rural towns; 6.54, 95% CI 4.25–8.84 in urban neighborhoods).

**Fig. 1:**
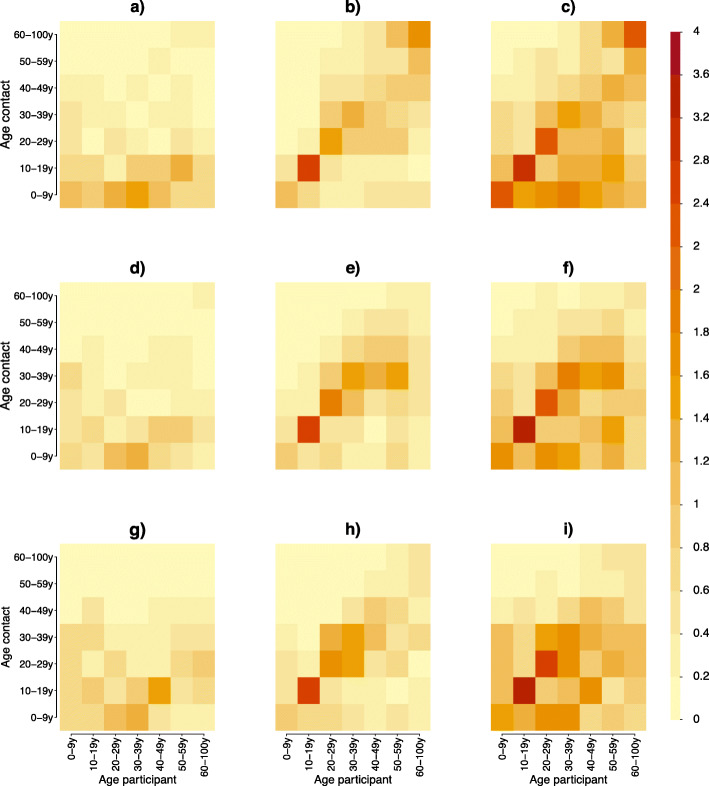
Contact matrix representing the mean number of daily contacts reported by a participant in the age group *i* with individuals in the age group *j* in household (**a**), in the general community (**b**), and both (**c**) in remote settlements. **d**–**f**, **g**–**i** The same quantities estimated for rural villages and for the urban neighborhoods, respectively

The analysis of contacts by age clearly shows that subjects below 30 years of age tend to interact mostly with individuals of similar age (assortative mixing). The highest contact rates were found between school aged children (10–19 years), between young adults (20–39 years), and between children below 10 years and their parents (Fig. 1, and Additional File 1: Sections 6 and 7). A marked intergenerational mixing both within households and in the community was found, especially in remote settlements.

The average overall number of daily contacts reported by our study participants (7.5 contacts), the share of contacts experienced with household members (46.1% including all ages), and the proportion of school contacts for children between 5 and 21 years of age (40.3%) are in line with estimates obtained by similar studies conducted in Zimbabwe, Uganda, and Kenya [19, 20, 34], where the number of contacts per day was found in the range 7–11, the proportion of contacts at home was 50–66%, and around 50% of contacts of school-aged children were recorded between schoolmates. The potential high level of mixing between the elderly and both young adults and children has been already highlighted for Ethiopia by the synthetic contact matrices estimated in Prem et al. [35].

### Effect of demography and age-specific contacts on COVID-19 epidemics

From the epidemic curve of reported cases, we estimated a net reproduction number *R* of 1.62 (95% CI 1.55–1.70) over approximately 6 weeks of exponential growth starting from May 1, 2020, when schools were closed in the entire country (see Additional File 1: Section 4). We relied on this estimate of *R* to simulate COVID-19 epidemics in the SWSZ considering no school contacts. If school contacts are included, we estimate *R* to increase up to 3.15 (95% CI 2.22–4.20, see Additional File 1: Section 4), which is comparable with estimates of the basic reproduction number from other parts of the world [36–39].

**Fig. 2:**
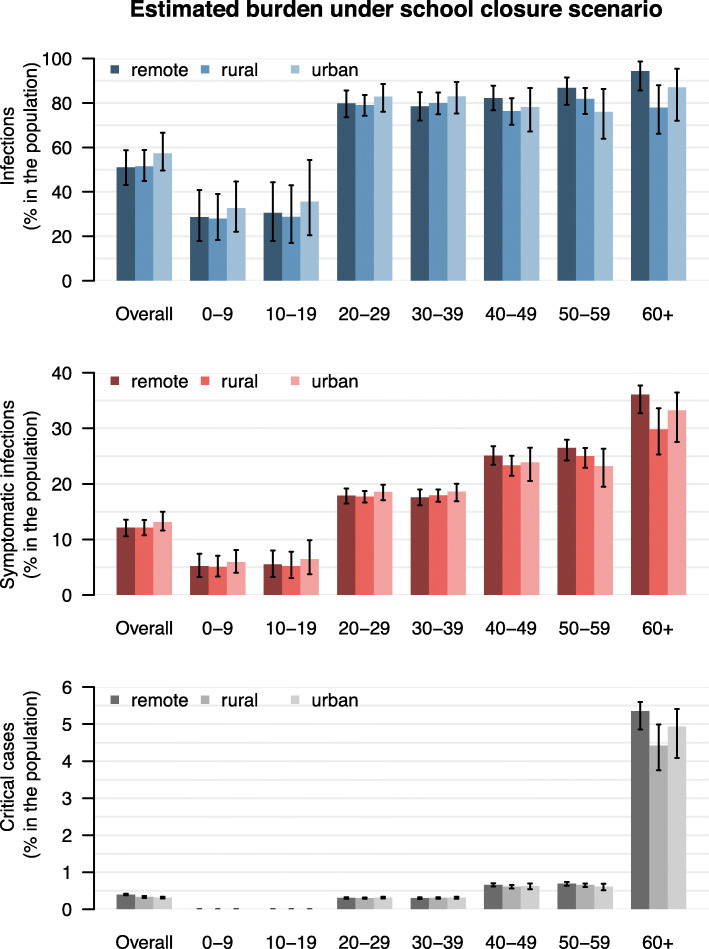
Estimated attack rates of infection (**a**), symptomatic cases (**b**), and critical disease (**c**), overall and by age group in different geographical contexts of the SWSZ, as expected at the end of an epidemic mitigated by school closure alone. Outputs were obtained by simulating 1000 different epidemics where the per-contact transmission rate is set to reproduce, when neglecting contacts occurring at school, random samples of the distribution of the net reproduction number estimated from national surveillance data: 1.62 (95% CI 1.55–1.70) [5]. Black lines represent 95% credible intervals

Our simulation results show that, had schools remained closed for the entire duration of the epidemic and had no other interventions been enacted, 12.1% (95% CI 10.8–13.5), 12.1% (95% CI 10.6–13.6), and 13.1% (95% CI 11.6–15.0) of the population residing in rural, remote, and urban settings respectively would have developed respiratory symptoms or fever because of COVID-19. The fraction of critical cases (requiring mechanical ventilation and/or resulting in a fatal outcome) is estimated between 0.28% and 0.41% of the overall population (Fig. 2). The highest prevalence of critical cases (between 4.4% and 5.4% on average) is expected within subjects aged 60 years or older. This age segment represents only about 5% of the total population in SWSZ but is expected to represent 7 to 14% of symptomatic cases and 43 to 63% of all critical cases.

Remote settlements are expected to suffer a higher overall burden of critical cases (0.40% of the total population, 95% CI 0.37–0.41%) compared to rural villages (0.33%, 95% CI 0.31–0.35%) and urban neighborhoods (0.31%, 95% CI 0.29–0.33%). This difference is explained by a higher proportion of the elderly in the population (see Additional File 1: Sections 6 and 7), but also by their higher number of daily contacts and the higher intergenerational mixing (Fig. 1c) compared to the other settings, which results in a higher attack rate of infections, symptomatic cases, and critical disease in this age group (Fig. 2). Urban neighborhoods, where the highest contact rates at younger ages were recorded, are expected to have the highest attack rate of infections (57.3%, 95% CI 49.6–66.7) and symptomatic cases (13.1%, 95% CI 11.6–15.0). However, since a large proportion of the overall number of infections (81.8%, 95% CI 76.1–85.3) is concentrated on children and younger adults (up to 40 years of age), this does not result in a high overall proportion of critical disease. Finally, rural villages have lower attack rates among the elderly because of the significantly lower number of contacts reported by that age group in this geographical context (Fig. 1f, Table 2).

**Fig. 3:**
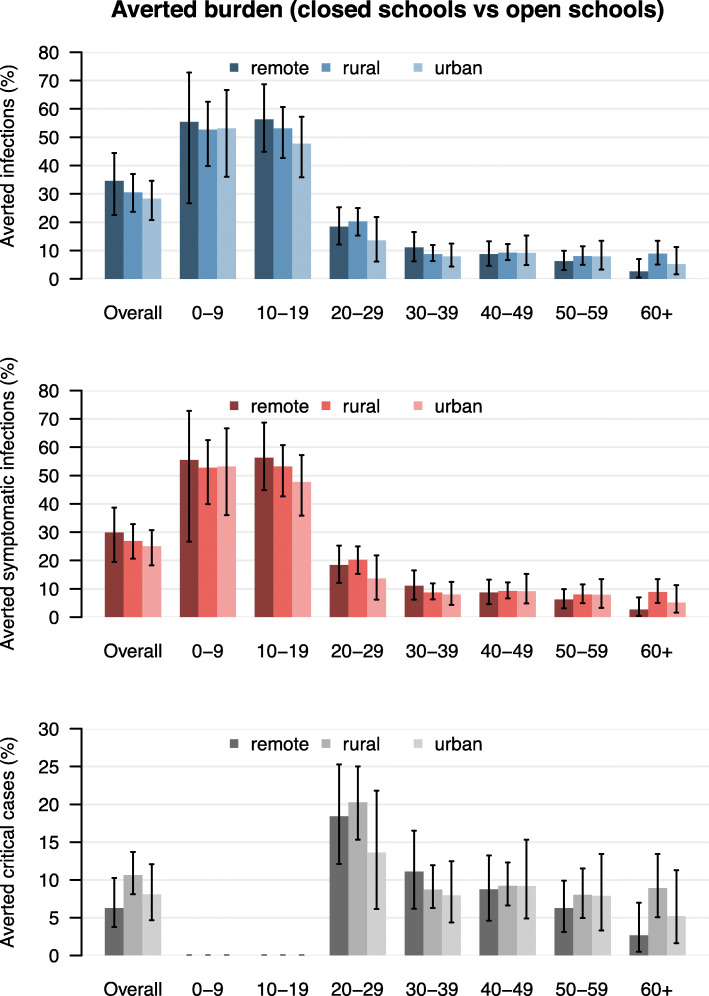
Estimated percentage of **a** averted infections, **b** symptomatic infections, and **c** critical cases, overall and by age group in different geographical contexts of the SWSZ with respect to a hypothetical scenario without school closure. Black lines represent 95% credible intervals

Figure 3 shows the impact of maintaining the school closure mandate on the burden of COVID-19 in the SWSZ in terms of percentages of infections, symptomatic, and critical cases averted with respect to a hypothetical scenario of an unmitigated SARS-CoV-2 epidemic. According to our estimates, the beneficial impact of school closure would consist of 26.9% (95% CI 20.7–32.8), 29.9% (95% CI 19.5–38.7), and 25.1% (95% CI 18.2–30.8) averted symptomatic cases and 10.6% (95% CI 8.1–13.7), 6.3% (95% CI 3.8–10.3), and 8.1% (95% CI 4.7–12.1) averted critical cases respectively in rural, remote, and urban contexts. As expected, the larger effect of the intervention in terms of averted infections is observable in younger ages, while the indirect effect of school closure on the elderly is highlighted by the expected high fractions of averted critical cases among individuals aged 50 or over.

**Fig. 4:**
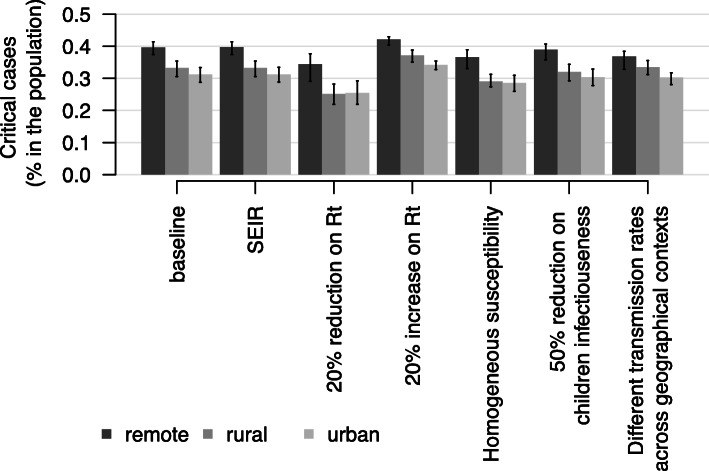
Comparison of the estimated overall percentage of critical cases in different geographical contexts of the SWSZ in the baseline and sensitivity analyses. Black lines represent 95% credible intervals

A comparison of model estimates obtained in our baseline analysis with those obtained in the sensitivity analyses is provided in Fig. 4. These results suggest that our estimates on the overall fraction of critical cases expected by maintaining the school closure mandate are robust against all alternative assumptions considered, ranging between 0.25–0.37%, 0.23–0.42%, and 0.25–0.34% for rural villages, remote settlements, and urban areas, respectively.

## Discussion

Our analysis explored the effect of demographics and social contact patterns on COVID-19 burden in the South West Shewa Zone of the Oromia Region, Ethiopia. Data collected with an interview-based survey highlighted differences in demographic structure and in age-specific contacts between urban neighborhoods, rural villages, and remote settlements and were used to inform an epidemic model simulating the transmission dynamic of SARS-CoV-2. On the basis of the trajectory of COVID-19 cases observed in the country up to June 12, 2020, we estimated that between 3.1 and 4.0 patients per 1000 inhabitants may experience critical disease (i.e., requiring mechanical ventilation and/or resulting in a fatal outcome) at the end of an epidemic mitigated by school closure alone. Considering the low availability and accessibility of healthcare, especially in remote and rural settlements, and the lack of intensive care units to treat critical patients [2, 40], it is possible that a large fraction of those cases would result in a fatal outcome, adding up to the already high background mortality rate in the region (estimated at about 6.4 per 1000 per year [41]).

Considering the extreme scenario where all critical cases would result in a fatal outcome, we obtain an estimate of the infection-fatality ratio (IFR) ranging between 0.55% in urban neighborhoods and 0.78% in remote settlements. Such estimates are generally lower than the IFR estimated from serological studies for higher income countries [42, 43]. This difference is partially due to the younger age structure of the Ethiopian population, where only 5% of individuals are older than 60 years (compared to over 20% in most of Europe [44]). However, by simply adjusting the age-specific IFR to the local demographics, Ghisolfi et al. [45] estimated a fourfold reduction in the overall IFR in Eastern Africa with respect to European countries, which is around 2 times lower than our estimates. In fact, our simulations not only account for the demography of the population, but also for its mixing patterns. Indeed, we found that in the SWSZ the effect of a younger population is partially compensated by high infection attack rates in the elderly, which derive from the intense intergenerational mixing and the larger number of contacts observed among the elderly. In particular, we show that these characteristics are especially marked in remote settlements, where the highest incidence of critical disease is expected to occur. Although our analysis is limited to the SWSZ, we expect that similar arguments may be generalizable to settings with similar socio-demographic conditions.

Our results suggest that, in the SWSZ, school closures might have reduced by 48.9% the SARS-CoV-2 reproduction number and by 28.3–34.6% the infection attack rate that would have been expected in the absence of any intervention. In line with observations from other settings [23], school closure was estimated to be insufficient to prevent the spread of the infection. Recently published studies have shown that the lockdown implemented in Kenya reduced individuals’ social interactions by 60–70% compared to the pre-pandemic period [15], but it is difficult to extrapolate these data to Ethiopia, where social distancing measures were comparatively milder. Data on how contacts outside school may have changed in Ethiopia during the COVID-19 epidemic are still lacking.

To properly interpret the results presented in our study, it is important to consider the following limitations. First, the target study population may be not representative of all Ethiopia and in particular of epidemic patterns observed in highly urbanized areas such as the capital Addis Ababa. Second, the net reproduction number was estimated from national surveillance data [5]. This data reports cases aggregated at the country level and may suffer from a number of biases: it does not account for reporting delays; the growth over time in the number of cases may partly be ascribable to the increase in testing capacity; total cases represent the superimposition of different, asynchronous epidemics in multiple parts of the country, a majority of which coming from the highly urbanized Addis Ababa area [9]. More in general, estimates of time-varying reproduction numbers from data where the symptoms’ onset time-series is approximated with the notification date series may inaccurately describe the early infection dynamics and could fail in assessing the impact of containment measures. However, we show that, when assuming no restriction to school contacts, the reproduction number estimated by the model is in the range 2.43–3.52, comparable with estimates of the SARS-CoV-2 basic reproduction number from other countries [36–39]. Moreover, our conclusions remain robust when considering a 20% increase or a 20% decrease of the reproduction number. In this case, we estimated an attack rate of critical cases ranging from 0.25 to 0.37 for rural villages and from 0.34 to 0.42 for remote settlements (see Fig. 4). Third, the model lacks spatial structure. The finding from the survey that about 97% of recorded contacts have occurred within the participant’s neighborhood of residence (Table 2) suggests that local containment or confinement of COVID-19 outbreaks in rural regions of Ethiopia may be favored by low human mobility. On the other hand, the observation of a large number of cases in all regions of Ethiopia [9] may imply that a significant widespread diffusion of the epidemic, possibly sustained by a high fraction of asymptomatic infections (Fig. 2), is ongoing. Fourth, the role played by children in the transmission of SARS-CoV-2 infections is still poorly understood and highly debated [23, 46]. In the main analysis, we assumed that the probability of transmission is homogeneous across all ages. As asymptomatic infections are more prevalent at younger ages, this also reflects the assumption that symptomatic and asymptomatic cases are characterized by the same infectiousness. However, an alternative assumption in which children are assumed half as infectious as adults would result in similar attack rates of critical cases (see Additional File 1: Section 8). These results are also robust with respect to the assumption of a homogeneous susceptibility across age groups (see Additional File 1: Section 8). Finally, in absence of direct data from sub-Saharan Africa, the age-specific susceptibility and proportions of infections resulting in symptomatic cases or critical disease were estimated from data from China or Europe [23, 32]. However, the high prevalence of comorbidities which are uncommon in higher income countries (e.g., malnutrition [47], tuberculosis, and malaria) and inequalities in the access to primary care represent additional vulnerabilities for African settings [2] and may result in an underestimation of the expected disease burden. Since the number of COVID-19-related deaths may be under ascertained in low-income countries, further research is warranted regarding the disease severity in sub-Saharan populations, potentially leveraging excess mortality data once they will become available.

## Conclusions

This study provides novel data on mixing patterns in rural Ethiopia and highlights the potential impact of COVID-19 epidemics in less urbanized regions of the country. We provide estimates on the potential burden of COVID-19 in the SWSZ under the assumption of a mitigated, but not controlled epidemic. We conclude that, although the overall mortality might be generally lower in sub-Saharan Africa compared to high-income settings, thanks to younger demographics [45, 48, 49], this effect may be partially offset in rural areas by higher attack rates in elderly individuals, due to high rates of intergenerational mixing. The observed contact patterns suggest that elderly individuals in remote settlements may be even more exposed to the risk of infection (and thus of critical disease), which is especially worrisome in light of the major obstacles in access to healthcare for those populations [2].

## Supplementary Information

**Additional file 1.** Appendix.

**Additional file 2.** Questionnaire used for conducting the contact study.

## Data Availability

Data analyzed during this study will be included in this published article as supplementary information files.
