# Supplementary material for: Modeling the interplay between demography, social contact patterns, and SARS-CoV-2 transmission in the South West Shewa Zone of Oromia Region, Ethiopia
Source: BMC Med. 2021 Apr 9;19:89. doi: 10.1186/s12916-021-01967-w (PMC8032453; doi:10.1186/s12916-021-01967-w)
Supplement: Supplementary file 2 — Additional file 2. Questionnaire used for conducting the contact study. [file 12916_2021_1967_MOESM2_ESM.pdf]

Questionnaire used for conducting the contact study

PART ONE: SOCIO-DEMOGRAPHIC CHARACTERISTICS

1. **In the first part, the interviewer is asked to fill in the ID of the participant, place and date of the interview.**

   1.1. *Individual participant ID:* |__|__|__|__|__|

   1.2. *Interview date (dd/mm/yy)* : |__|__|__|__|__|__|__|

   1.3. *Address of the household (Woreda/Kebele) :* |_________________|_________________|

2. **We would first like to ask a few questions about yourself/your child as well as about the people living with you/your child in your household.**

   2.1. *Including yourself/your child, could you please list all individuals in your household, as well as the family links between yourself/your child and each of the household members.* ***[A household is the group of individuals living under the same roof and sharing the same kitchen on a daily basis]***

Household member initials/name	Household member ID			Sex<br><br>M=1<br>F=2	Age in years	Relationship						
						me	Spouse	Sibling	Child	Parent	Other family	Unrelated
	H	0	1			*X*						
	H											
	H											
	H											
	H											
	H											
	H											
	H											
	H											
	H											
	H											
	H											
	H											
	H											

2.2. *What is your/your child's primary occupation or daily activity [note to the interviewer: this is the activity the participant spends the most time doing on a daily basis]?*

pre-school child		*agriculture*		*retired*	
student (any school)		*manual worker*		*others*	
office worker		*housewife*		*don't know*	
shop worker		*unemployed*		*refused*	

2.3. *If the participant is a student,*

2.3.1. *Where is her/his school (Woreda/Kebele)? |________________________________*

|/|________________________________|

2.3.2. *What is her/his grade? |_________ |*

2.3.3. *How many students are there in her/his class? |_________ |*

2.4. *If the participant is a worker,*

2.4.1. *Where is her/his place of work (Woreda/Kebele)? |___________________________*

|/|__________________________________|

2.4.2. *How many individuals are there in her/his workplace? |_________ |*

PART TWO: CONTACTS and MOBILITY

1. **We would like to ask a few questions about your/your child's contacts on the day before the interview.**

1.1. *We will first ask a few more questions about the people you were/your child was in contact with yesterday excluding contacts at school (for both students and teachers)* ***[A contact is here defined as an interaction between two individuals and it can be either: 1) physical: involving a skin-to-skin contact, such as a handshake or hug; 2) non-physical: involving a two-way conversation with five or more words in the physical presence of another person (standing at most at two-arm distance), but no skin-to-skin contact. Yesterday is defined as the period from wake up yesterday to the moment you woke up this morning]***

Contact initials/name [will be removed from the questionnaire after the interview]	Contact ID			Place ID			Age (yrs)	Sex (M/F)		Type of contact		Link to the contact					Frequency	
								M	F	Physical	Nonphysical	Household member	Other relative	colleague or schoolmate	Friend	Other	Once	More than once
	C	0	1	S	0	1												
	C			S	0	1												
	C			S	0	1												
	C			S	0	1												
	C			S	0	1												
	C			S	0	1												
	C			S	0	1												
	C			S	0	1												
	C			S	0	1												
	C			S	0	1												
	C			S	0	1												
	C			S	0	1												

1.2. If the study participant is a student or a teacher
How many physical contacts has she/he had yesterday at school?

2. We would like to ask a few questions about your/your child's travels outside the Kebele of residence.

2.1.1. Specify the name of the Kebele of the furthest place you/your child visited in the last month: |________________________________|

2.1.2. How many times did you travel to this place? |_______|

2.1.3. How long did you/your child stay for on average (in days)? |_______|

2.1.4. When was you/your child's last visit to an HC?

Never		< 1 week		*2-4 weeks*		*> 1 month*	

2.1.5.Has you/your child's been admitted to Saint Luke Hospital?

Never		< 1 week		*2-4 weeks*		*> 1 month*	

3. In this questionnaire, the interviewer will ask the school director some information about the school.

3.1. *School ID:* |__|__|__|__|

3.2. *Address of the school (Woreda/Kebele) :* |_________________|_________________|

3.3. *We will now ask a few questions about the structure of the school.*

**ID class**			**Grade**	**Students' Minimum age**	**Students' Maximum age**	**Number of students**	**Number of teachers**
CL	0	1					
CL	0	2					
CL	0	3					
CL	0	4					
CL	0	5					
